# Is It State Aid?

**Published:** 1920-04-24

**Authors:** 


### The Hospital

The Workers' Newspaper of Administrative Medicine and Institutional Life, Administration, National Insurance and Health.

No. 1768, Vol. LXVIII. SATURDAY, APRIL 24, 1920. PRICE TWOPENCE

### IS IT STATE AID?

The Parliamentary Correspondent of *The Times* is responsible for the statement that the Government are considering whether they cannot include further provision for the hospitals of the country in the Health Services Bill, which the Ministry of Health is now drafting for presentation to Parliament in a few weeks' time.

The Ministry have already approved of the provision of a municipal general hospital at Bradford, and the proposal now under examination is that this action, thought by some to be *ultra vires*, may be definitely sanctioned by legislation, local authorities being given power to establish new hospitals where they are needed. It is admitted that there are great gaps in hospital provision and these cannot immediately be filled by the present voluntary system, battling to hold its own; although in the expansive power of that system, momentarily affected by new and adverse conditions, we have every confidence. That the voluntary hospitals will, given vigorous management, win through we do not doubt for an instant, but an extension of the system on a sufficiently large scale is not possible at the present time. Government action, therefore, to supply public needs appears necessary as a supplementary service. We are informed that the Ministry of Health feel that this is particularly the case in respect of London. Power will also be sought to link existing institutions with the local health services and to make further and better general provision, especially for women and children. Clinics will be established to diminish the number of those who now go straight into hospital.

Certainly this is an opportunity for the presentation of a concerted scheme of health service, bringing into affiliation the voluntary hospitals, the municipal infirmaries, and new municipal hospitals, infant and maternity centres, district nursing, and other appropriate forms of institutional and home medical aid and nursing. It goes without saying that the new Act could only be a framework upon which detailed parts of the great scheme will find their proper place at the proper time, a scheme which will be built up with care, in the light of experience and without undue hurry. One important point will be how and when the municipal or voluntary hospital will take care of the insured person and on what terms such assistance will be given. How far the discharged soldier will enter into the scheme it is hard to say, but this class of patient will be with us for many years to come, and unless the Ministry of Pensions are able entirely to take charge of him, in their own institutions, presumably, he will come within a Health Services Act.

The financial position of the voluntary hospitals of London has now reached a very acute stage. Within the last few days the chairmen of about twelve of the largest Metropolitan hospitals met and waited upon Dr. Addison with a request for immediate relief from the Ministry of Health, the position being that in many cases the hospitals have been refused further loans from the banks. As we have said, we have every confidence that the voluntary hospitals can and will survive the storm, but it is simply our present duty to record facts. State help has at last been asked for, even if it be only temporary State help.

We believe we are right in saying that shortly before the time of his death Sir Robert Morant was engaged upon a scheme of alliance between the Health Ministry and the hospitals, under the terms of which a certain part of any deficit of an institution would, after accounts had been approved, have been made up from Government funds. Now, we understand, several alternative methods of help have been discussed, one being the payment of one-third of approved expenditure, another a *pro rata* augmentation of charitable gifts.

Dr. Addison, however, is as steadfast as ever in his view that there can be no question of the hospitals being "taken over," and there is every indication that though these institutions are passing through very troubled waters they will weather the storm and keep the voluntary flag flying for years to come.

